# A Preliminary Study to Investigate the Genetic Background of Longevity Based on Whole-Genome Sequence Data of Two Methuselah Dogs

**DOI:** 10.3389/fgene.2020.00315

**Published:** 2020-04-16

**Authors:** Dávid Jónás, Sára Sándor, Kitti Tátrai, Balázs Egyed, Enikö Kubinyi

**Affiliations:** ^1^Department of Ethology, ELTE Eötvös Loránd University, Budapest, Hungary; ^2^Department of Genetics, ELTE Eötvös Loránd University, Budapest, Hungary

**Keywords:** extreme longevity, aging, dog, genomics, whole genome sequence

## Abstract

Aging is the largest risk factor in many diseases and mortality alike. As the elderly population is expected to increase at an accelerating rate in the future, these phenomena will pose a growing socio-economic burden on societies. To successfully cope with this challenge, a deeper understanding of aging is crucial. In many aspects, the companion dog is an increasingly popular model organism to study aging, with the promise of producing results that are more applicable to humans than the findings that come from the studies of classical model organisms. In this preliminary study we used the whole-genome sequence of two extremely old dogs – age: 22 and 27 years (or 90–135% more, than the average lifespan of dogs) – in order to make the first steps to understand the genetic background of extreme longevity in dogs. We identified more than ∼80 1000 novel SNPs in the two dogs (7500 of which overlapped between them) when compared to three publicly available canine SNP databases, which included SNP information from850 dogs. Most novel mutations (∼52000 SNPs) were identified at non-coding regions, while 4.6% of the remaining SNPs (n∼1600) were at exons, including 670 missense variants – 76 of which overlapped between the two animals – across 472 genes. Based on their gene ontologies, these genes were related – among others – to gene transcription/translation and its regulation, to immune response and the nervous system in general. We also detected 12 loss-of-function mutations, although their actual effect is unclear. Several genetic pathways were also identified, which pathways may be tempting candidates to be investigated in large sample sizes in order to confirm their relevance in extreme longevity in dogs (and possibly, in humans). We hypothesize a possible link between extreme longevity and the regulation of gene transcription/translation, which hypothesis should be further investigated in the future. This phenomenon could define an interesting direction for future research aiming to better understand longevity. The presented preliminary results highlight the utility of the companion dog in the study of the genetic background of longevity and aging.

## Introduction

Aging is considered as one of the largest risk factors for both diseases and mortality (Flatt and Partridge, 2018). Age-related diseases include sensory changes (e.g., hearing loss), systemic (e.g., high blood pressure) and neurodegenerative disorders (e.g., Alzheimer’s disease) among others (Jaul and Barron, 2017). Furthermore, both the number and proportion of elderly people are expected to increase at an accelerating rate in the future (the United Nations estimated a 230% increase – from 901 million to 2.092 billion – in the population of people of 60 years or above from 2015 to 2050; United Nations, 2015). Both longevity and healthspan (defined as the “period of time during which humans and non-human animals are generally healthy and free from serious or chronic illness”; quote from Wallis et al., 2018) are direct consequences of (a healthy) aging and are equally important from both social and economic points of view.

The genetics of aging and longevity has been studied in multiple species, including *C. elegans* (e.g., Gems and Riddle, 2000), fruit fly (e.g., Lehtovaara et al., 2013), mice (e.g., Piper et al., 2008), dogs (see Hoffman et al., 2018 for a summary), and humans (e.g., Herskind et al., 1996). Based on these studies, longevity is known to be influenced by both genetic and environmental factors (López-Otín et al., 2013), with an estimated heritability of 15–30% in humans (e.g., Herskind et al., 1996); in a comparative study, Yanai et al. (2017) synthesized the results currently available on longevity-associated genes (LAGs) from yeast, *C. elegans*, fruit fly and mouse and studied their orthologs in more than 200 species. More recently, a study showed that heritability of lifespan might have been overestimated in the past and an upper limit estimate of ∼7% was proposed (Ruby et al., 2018). However, extreme longevity (i.e., longevity of centenarians) was reported to have a higher heritability than longevity itself (Sebastiani et al., 2016). Several mechanisms of aging are either directly (genomic instability, telomere attrition, and epigenetic alterations) or indirectly related to genetics [cell senescence (Yanai and Fraifeld, 2018), loss of proteostasis and stem cell exhaustion (López-Otín et al., 2013)]. Furthermore, multiple genetic pathways have already been identified to be linked to longevity, such as the insulin/insulin-like growth factor signaling pathway, the telomere maintenance pathway or the DNA damage response and repair machinery (Deelen et al., 2013; Moskalev et al., 2013; Debrabant et al., 2014). All of these pathways are crucial to sustain normal cell functions and are related to the previously mentioned genetic hallmarks.

For many reasons, the companion dog is an especially promising model organism for translational approaches in aging research (Gilmore and Greer, 2015; Kaeberlein et al., 2016; Sándor and Kubinyi, 2019). Firstly, dogs have a much shorter life expectancy than humans – 10–13 years on average in dogs (Adams et al., 2010; Leroy et al., 2015; Inoue et al., 2018) vs. 72 years in humans (2016 estimation; World Health Organization [WHO], 2018) – making aging-related longitudinal studies much shorter in time. Secondly, dog breeds show a huge phenotypic and genetic variability (Leroy et al., 2009; Ostrander et al., 2017), which can also be observed in longevity (e.g., Jimenez, 2016; Inoue et al., 2018). Dogs also have more shared haplotype sequences with humans than rodents do (Lindblad-Toh et al., 2005). Furthermore, they also experience the rich and variable environment and lifestyles their human owners live in (unlike laboratory animals, which are kept in a highly controlled environment) and therefore similar environmental factors influence their everyday life, aging and life expectancy (Hoffman et al., 2018) – albeit, at least two major exogenous factors affecting longevity (namely, diet and physical activity) may be considerably different between the two species.

Finally, dogs are prone to multiple, spontaneously developing age-related diseases late in their lives that can be considered as analogs of multiple human diseases (e.g., the canine cognitive dysfunction, which is similar to Alzheimer’s disease; see Chapagain et al., 2018 for more details). The undeniable relevance of this final point and the previously mentioned other advantages of the companion dog make this species one of the best model animals to study aging. In addition, it is also expected that the obtained results will be more applicable to humans than those conducted on canonical laboratory animals. In a review article, Sándor and Kubinyi (2019) summarized our state of the art knowledge about the genetic pathways involved in aging in dogs.

The study of aging and longevity is of great importance. It enables us to better understand the process of aging itself, which in turn could lead to the promotion of a healthy lifestyle among the general population and help mankind to successfully cope with the long-term socio-economical consequences of an aging population. In addition, a deeper understanding of extreme longevity and its background is fundamental for biomedical research to develop efficient medicine against age-related diseases (both for treatment and prevention). Furthermore, it was highlighted before in humans, that the prevalence of multiple age-related diseases was lower among the offspring of centenarians and that longevity-related genes provide a certain level of ‘protection’ against cognitive decline and neurodegeneration (e.g., Sanders et al., 2010 after Han et al., 2013). However, at this point it is worth mentioning that genomes of centenarians also contain buffered deleterious alleles (e.g., Huffman et al., 2012). Moreover, in dogs, the identification of genes and other genetic loci linked to longevity would enable breeders to select more efficiently for longevity within the breeds. As a result of selection on such genetic loci in dogs, the proportion of the beneficial alleles could be increased within the breed under selection, increasing the average life expectancy of the given breed and simultaneously improving the quality of life of the companion pets and their owners alike.

Han et al. (2013) studied six centenarians (105–109 years old) to investigate the genetic background of extreme longevity in humans. These centenarians lived ∼50% longer compared to the average human lifespan (72 years, World Health Organization [WHO], 2018). Given this definition of extreme longevity and the average lifespan of companion dogs (10–13 years; Adams et al., 2010; Leroy et al., 2015; Inoue et al., 2018), dogs older than ∼17 years can be considered as dogs of extreme age. Mixed-breed dogs are known to live longer: Inoue et al. (2018) studied the lifespan of more than 12,000 dogs and found the average length of lifespan of mixed-breed individuals to be 15 years. Therefore, extreme longevity in their case corresponds to ∼22.5 years of age. In the data published by Inoue et al., 13 dogs lived 22–25 years (no dogs above the age of 25 were recorded in their study), corresponding to 0.1% of the studied population, or 1.16% of the mixed-breed individuals, assuming that all individuals of age 22–25 were mixed-breed. These numbers suggest that there is a sufficiently large population of dogs with an extreme longevity to be included in age-related studies in the future.

In this preliminary study we analyzed the whole genome sequence of two extremely old dogs, who lived 22 and 27 years – or approximately 50–80% longer, than the average lifespan of a mixed-breed dog (alternatively, 90–135% longer, than the average canine lifespan). Our primary aim was to investigate the genetic background of longevity and identify mutations at coding regions that could have contributed to their long lifespan. Our secondary aims were to compare the obtained results to the results of Han et al. (2013), to extend our understanding of extreme longevity and to promote the companion dog to be used in age-related research. Indeed, this is the first such study in canines, although based on the results of Inoue et al. (2018), Methuselah dogs are available for the study of aging and longevity.

## Materials and Methods

The canine reference genome (*CanFam 3.1* version), as well as all relevant information related to it (e.g., gene annotations) were downloaded from Ensembl (version 94, released in October, 2018; Hunt et al., 2018). Since the canine reference genome excludes the Y-chromosome, this chromosome was not included in the analysis.

### The Dogs Under Analysis

**FIGURE 1:**
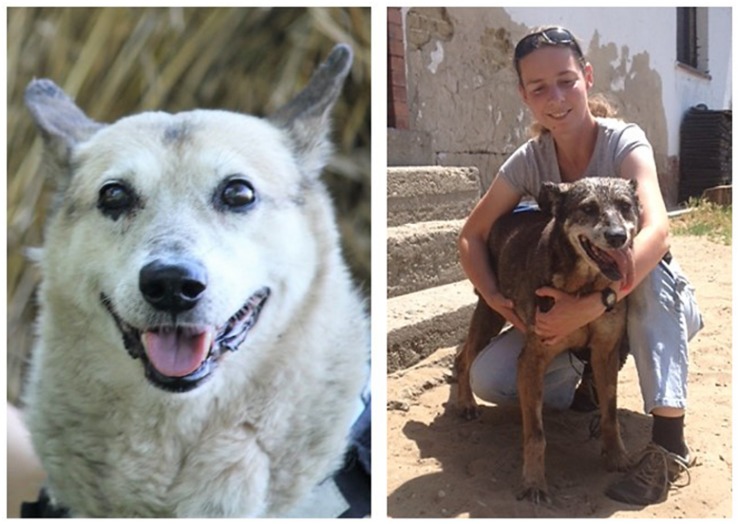
The two Methuselah dogs participating in this study: Buksi **(left)** (photo taken by Attila Dávid Molnár) and Kedves **(right)** (photo taken by Linda Gerencsér). The images are published with the written consent of owners and all identifiable person(s).

Two dogs of extreme age (i.e., Methuselah dogs) were examined in this study: a 27 years old mixed-breed intact male (Buksi, lived in Sárrétudvari, Hungary; ID: old_rep1; buccal swab sample collected at the age of 26) and a 22 years old mixed-breed neutered female dog (Kedves, lived in Ócsa, Hungary; ID: old_rep2; blood sample collected at the age of 22, Figure 1). The distance between the living areas of the dogs was approximately 180 km. Both dogs lived in a rural environment in Hungary where they were allowed to roam around freely. Therefore, both of them could feed on wild prey animals (e.g., mice) beyond their daily meals. The male dog had a diet of raw chicken and table scraps and had been fed this way through his whole life, according to the owners. He lived in a horse riding center and had contact with friendly people (both unfamiliar and familiar) who went for horse riding on a daily basis, as well as with other dogs at the farm and in the village. The female dog was fed with commercial dog food *ad libitum*. She was a pet dog of a dog shelter manager, and also had plenty of contacts with people visiting the shelter, the management staff and other dogs. Both animals were in good physical condition at the time of sampling. The male dog weighted around 13–14 kg, while the female dog weighted around 16–18 kg. Both of them were regularly vaccinated against rabies.

Both dogs spent their entire life with their owners and their family, thus at least five adult persons – including the dogs’ veterinarians – who had no conflict of interest, confirmed the age of each dog.

### Whole-Genome Sequence Data

DNA samples were isolated and sequenced by Omega Biosciences (Norcross, GA, United States). Sequencing was performed on an Illumina HiSeq X Ten instrument, producing 150 basepairs long paired-end reads. A total of ∼2 × 481 and ∼2 × 473 million reads were sequenced for the two samples.

### On-Line Databases

Our working hypothesis was that common variants (i.e., variants segregating in dogs with an average lifespan) were less likely to positively affect longevity than variants uniquely present in individuals with extreme longevity. Therefore, our primary focus was on the short genetic variations that are uniquely present in the Methuselah dogs sequenced within the framework of this study and are absent in dogs with an average lifespan. In order to exclude the most common variants, all SNPs and indels previously identified and published in at least one of three on-line databases were excluded. These databases included: (1) the Dog Genome SNP Database (DoGSD), which is “a data container for the variation information of dog/wolf genomes” (quote from the DoGSD website accessed on 25/06/2019^1^) and contained SNP data from 127 individual samples, including purebred and mixed-breed individuals alike; (2) the database created by the [American] National Human Genome Research Institute (NHGRI) based on the whole-genome sequence of 722 dogs; (3) the Broad Institute’s dog SNP database, which was created as part of the Canine Genome Sequencing Project. The last database was created for the *CanFam 2.0* genome version and therefore positions were lifted over to the current genome version (*CanFam 3.1*), which was used in this study; 16,388 SNPs (15,951 of which were on autosomes) were removed in the process, out of the 2,544,508 from the original study (Table 1).

**TABLE 1:** Number of SNPs in three, previously published databases.

Database	38 autosomes + X chromosome^1^	38 autosomes
DoGSD database (Bai et al., 2015)	54644335	52318004
Broad Institute database (Lindblad-Toh et al., 2011)^2^	2528120	2466855
NHGRI database (Plassais et al., 2019)	20269614	19693593
Total number of non-redundant SNPs	61180804	58623548

*^1^+MT in case of the DoGSD database. ^2^After lift-over from CanFam v2.0 to v3.1.*

All animals in these databases were considered to be of average age. Although, the exact age of death of these individuals is unknown and therefore there might be some misclassification of these individuals. However, Inoue et al. (2018) studied the cemetery data of more than 12000 dogs and found that only 0,1% of them (*n* = 13) lived 22–25 years of age and none lived for 25 + years. Therefore, the probability of misclassification of the ∼800 dogs in the database is negligible.

The NHGRI’s database included indel mutations as well (*n* = 12,300,815), which were used to remove the common indels. It is important to note that since indels were not included in two of the databases, the common indel variants were detected in a smaller pool of individuals and therefore indels that are otherwise common among dogs with an average lifespan might have remained in the dataset.

### Candidate Gene Set

In a previous study, Han et al. (2013) discovered 89 novel non-synonymous SNPs via exome sequencing in six centenarians by targeting a predefined set of 988 genes. These genes were selected from previous publications and were from pathways that are known to be involved in either aging or longevity. In this study we included an additional 157 genes that were related to autophagy and other pathways, which also may affect aging (adding up to 1,145 genes in total) and identified their canine homologs (in total, 1,062 homologs were found). Although the related genetic pathways were already associated with aging, only limited information is currently available regarding the contribution of individual genes to aging either in humans or in dogs (Sándor and Kubinyi, 2019).

### WGS-Data Processing

**FIGURE 2:**
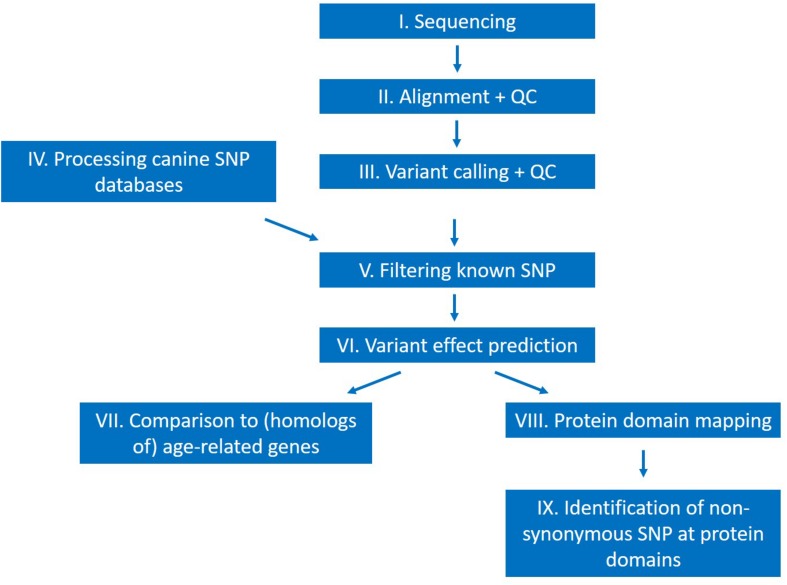
Outline of the performed study.

The general outline of the analysis is shown on Figure 2. Following sequencing, a quality control step of the raw reads using the FastQC program (Andrews, 2010; RRID:SCR_014583) was implemented. Alignment was performed with the *mem* command of the BWA aligner software (Li and Durbin, 2009; BWA, RRID:SCR_010910), using the standard parameter settings except for the “-M” option, which was used to make the output files compatible with the Picard software toolkit (Broad Institute, 2009; Picard, RRID:SCR_006525). Alignment quality control was assessed by calculating alignment statistics with Samtools (Li, 2011; SAMTOOLS, RRID:SCR_002105) and Picard.

Short variants (SNPs and short insertions-deletions) were then identified with the GATK software (Van der Auwera et al., 2013; GATK, RRID:SCR_001876). Short variants were called using the *HaplotypeCaller* command of GATK and separately for each chromosome to accelerate the variant calling step. The standard parameter values were used during variant calling as well, except for the number of allowed processors, which was increased from 1 to 8. Files containing the variants from different chromosomes were then merged with the *MergeVCFs* command (Picard) and the different types of variants (SNPs and indels) were separated with the *SelectVariants* command of GATK. This latter step was necessary, because different filtering options were applied for the different types of mutations.

Variants were filtered based on quality scores using the *VariantFiltration* tool (GATK). In case of both SNPs and indels, the recommended hard-filtering options were used. For SNPs, the applied filtering options were: QD < 2.0; FS > 60.0; MQ < 40.0; MQRankSum < 12.5; ReadPosRankSum < −8.0; SOR > 3.0. For indels, the recommended filtering options were used, except for the inbreeding coefficient, which parameter was excluded, as this option requires 10 or more individuals in the analysis (the applied filtering options in case of indels are: QD < 2.0; FS > 200.0; ReadPosRankSum < −20-0; SOR > 10.0). After quality-based variant filtration, the SNP and indel variants that were identified in both individuals were determined, as these variants are of greatest interest. The overlap category in the tables hereinafter will refer to this set of SNPs and indels.

### Downstream Analysis

All SNP and indel mutations published in at least one of the three canine databases were excluded from the analysis. Ensembl’s *Variant Effect Predictor* software (McLaren et al., 2016; Variant Effect Predictor, RRID:SCR_007931) was used to identify mutations with a potentially high impact on the phenotype and the genes incorporating one or more such mutations were identified. These genes were then compared with the age-related gene set defined above. In parallel, protein domain information was downloaded for all protein-coding genes from Ensembl for the *CanFam 3.1* genome version and non-synonymous SNP mutations located at these genomic regions were identified. The distribution of these SNPs as well as the function of the affected genes was investigated.

## Results

In spite of the similar sequencing depth between the two individuals, depth of coverage differed significantly between them after alignment to the reference genome. The average depth of coverage across the whole genome was 46.1 and 60.1 for old_rep1 and old_rep2, respectively.

Table 2 shows the number of SNPs and indels identified in this study before and after filtering out the previously published mutations (*n* = 61,180,804; see Table 1). The two Methuselah dogs were very similar in these numbers and a large proportion of both SNPs (64%) and indels (52%) overlapped between them. The number of unpublished SNPs was also similar between the two individuals (1.4% of all detected SNP), but only ∼17% of those SNPs were shared between them (Table 2). Indels showed a similar picture as SNPs, except for the unpublished indels (on average 17% of the indels were novel) and their overlap between the two individuals (62%), which were considerably higher.

**TABLE 2:** Number of variants discovered in the two individuals with extreme longevity as well as the number of overlapping mutations.

	old_rep1	old_rep2	Overlap
Number of SNP	4754086	4817227	3038929
Number of SNP (autosomes)	4648426	4688907	2973573
Number of unpublished SNP	41099	46375	7505
Number of indels	552996	578712	295412
Number of indels (autosomes)	521840	556968	280773
Number of unpublished indels	97826	99698	62288

Most of the SNPs located in genes were found in introns (92%), while only a small proportion of them were located in exons (∼4.6%), out of which ∼2.4% were missense variants (Table 3; for a complete list of all categories, see Supplementary Table S1). The remaining SNPs were located in the 3′ and 5′ UTR regions (1,3 and 2%, respectively), and a small number of loss-of-function mutations (start codon lost, stop codon gained/lost mutations; ∼0.045% of the SNPs) were detected as well. Loss-of-function mutations were detected in 12 known genes, none of which overlapped between the two individuals:

**TABLE 3:** Number of SNPs for different annotation categories.

Annotation category	old_rep1	old_rep2	Overlap
5′ UTR variant	323	379	121
Start lost	1	4	0
Intron variant	15507	17277	2798
Missense variant	422	411	76
Synonymous variant	387	394	86
Stop gained	5	5	0
Stop lost	1	0	0
Stop retained variant	199	248	30
3′ UTR variant	323	379	121

*The table also includes the unknown genes.*

1. Three genes had a start codon lost mutation: ENSCAFG00000007858, ENSCAFG00000011630, and ENSCAFG00000030632.
2. Nine genes had a stop codon gain mutation in the gene body: ENSCAFG00000000433, ENSCAFG00000008061, ENSCAFG00000010498, ENSCAFG00000017946, ENSC AFG00000024414, ENSCAFG00000002007, ENSCAFG 00000004279, ENSCAFG00000029250, and ENSCAFG 00000032497.

Although intron variants usually do not affect protein expression levels directly, they might still influence gene expression by, e.g., modifying transcription factor binding sites. However, since no information was available about the location of transcription factor binding motifs on the canine genome, this could not be assessed in this study.

Table 4 shows the number of genes that include SNP variants in either exons or regulatory regions (defined as 5 kb upstream and downstream of every protein coding gene) for the two animals and the shared set of SNPs between the two dogs (the number of SNPs is also shown). 327 novel exonic SNPs were detected in both dogs, while an additional 2505 exonic SNPs were identified in either one of them. Among the 327 shared SNPs, there were 67 missense mutations falling in 37 genes. In total, 13,334 SNPs were identified in regulatory regions, 1861 of which were shared between the two individuals, failing in 887 protein-coding genes. 31 of these genes were included in the age-related gene set as well. However, the investigation of the potential effect of these SNPs is not feasible from WGS-data only.

**TABLE 4:** Number of SNPs located within known age-related genes and in their promoter regions.

	old_rep1	old_rep2	Overlap
Number of longevity-related genes in dogs^1^		1062	

Number of exonic SNP	86621	86521	55159
Number of novel exonic SNP	1371	1461	327
Number of novel, missense SNP mutations	376 (257)	361 (261)	67 (37)
Number of age-related genes with missense mutation(s)	9 (8)	16 (12)	1 (1)

Number of genes with novel SNP in regulatory regions^2^	7014 (4370)	8181 (5005)	1861 (887)
Number of age-related genes with mutations in regulatory regions^2^	411 (159)	464 (168)	200 (31)

*Number of affected genes are shown in parenthesis. ^1^The definition of longevity-related genes is provided in the materials and methods section; ^2^defined as 5 kb upstream and downstream of gene body.*

We previously identified from the literature a set of 1,062 genes that could possibly be related to aging and longevity. Out of these, 19 had novel missense mutations in the studied dogs, one of which (ENSCAFG00000014403) was found in both individuals. Based on its gene ontology (biological process category, inferred from electronic annotation by Ensembl), it is related to intracellular protein transport and long-term synaptic depression. A complete list of these 19 genes and their associated gene ontology IDs can be found in Supplementary Table S2. Three examples with associated gene ontologies are presented below:

1. ENSCAFG00000024527: ion transport, chemical synaptic transmission,
2. ENSCAFG00000001710: nervous system development, synaptic transmission – cholinergic, excitatory postsynaptic potential,
3. ENSCAFG00000018622: positive regulation of protein phosphorylation, leading edge cell differentiation, negative regulation of apoptotic process.

We also examined the possible effects of missense SNPs on the protein level. The missense SNPs located in protein domains were identified and evaluated across all protein-coding genes (see Supplementary Table S3 for a complete list of these genes and their human homologs). In total, 180 genes hosting 248 missense mutations were identified, out of which 19 SNPs at nine genes were shared between the two individuals. Interestingly, in the case of the four genes (ENSCAFG00000002184, ENSCAFG00000002366, ENSCAFG00000009496, ENSCAFG00000018626) that included multiple shared SNPs between the two Methuselah dogs, the missense SNPs were close to each other (within 3–60 bp). These SNPs and the functions of the affected genes are discussed in detail in the Discussion section. Three example genes with missense mutations in protein domains as well as with some of the related GO terms are listed below:

1. ENSCAFG00000003004: negative regulation of cell growth, negative regulation of apoptotic process;
2. ENSCAFG00000004892: regulation of apoptotic process, positive regulation of extrinsic apoptotic signaling pathway;
3. ENSCAFG00000019380: calcium ion transport, regulation of heart contraction, regulation of blood pressure, modulation of chemical synaptic transmission.

### Analysis of Short Insertions and Deletions

In the case of both individuals, 75% of the indels were 5 bp or shorter and 86% were shorter than 10 bp. Indels longer than 100 bp were extremely rare (∼0.36%). Table 5 shows the number of protein coding genes with indels, grouped by impact category. Surprisingly, 524 genes hosted at least 1 short insertion/deletion of a measurable effect, according to Ensembl’s *Variant Effect Predictor*. High impact categories in Table 5 included mutations such as stop codon lost mutations (leading to elongated transcripts), indels resulting in frameshift mutations or insertions/deletions affecting coding regions. This group was the most numerous from the three impact categories.

**TABLE 5:** Number of genes including at least 1 indel per impact category.

	Impact category^1^	Protein-coding genes	Protein-coding genes (excl. protein families)
old_rep1	High	367	56
	Moderate	85	27
	Low	1	0

old_rep2	High	397	60
	Moderate	95	27
	Low	0	0

*^1^Impact category groups were defined by Ensembl as follows: HIGH – The variant is assumed to have high (disruptive) impact in the protein, probably causing protein truncation, loss of function or triggering nonsense mediated decay; MODERATE – A non-disruptive variant that might change protein effectiveness; LOW – Assumed to be mostly harmless or unlikely to change protein behavior (https://m.ensembl.org/info/genome/variation/prediction/predicted_data.html).*

## Discussion

The study of aging and longevity holds the promise to increase human average longevity and healthspan. As it was pointed out earlier by multiple research groups (e.g., by Gilmore and Greer, 2015 in their article entitled “Why is the dog an ideal model for aging research?”), the companion dog has the potential to overcome some of the limits of laboratory animals in aging and longevity studies and lead to results that are more applicable to humans. Here, we present the first, preliminary study on extreme longevity in companion dogs and also pointing at genes that could have played a role in their extreme longevity and thus can be candidates for verification studies conducted on larger cohorts.

We observed approximately 4.8 million SNPs and 550 1000 indels in the two test subjects. A large part of these short genetic variants overlapped between the two samples. This can be explained by the origin of the reference genome: the dog reference genome is originated from a female boxer and therefore it is not surprising that the mixed-breed individuals analyzed in this study carried different alleles at many loci. Therefore, the majority of the SNP and indel variants (and most likely other types of genetic variants as well) were shared between our two old subjects. The difference in the depth of coverage between the individuals did not influence the variant calling, suggesting that even the lower coverage (46x) was sufficient to detect SNP and indel variants. This is in accordance with the literature, where ∼30x coverage was proposed to be sufficient for SNP and indel calling (Sims et al., 2014).

Compared to SNP mutations, both the total number of previously unpublished indels and their overlap between the two individuals were considerably higher. This is because more novel indels were detected (∼17% of all indels) than novel SNPs. This might be for several reasons: first, only one of the three databases used to filter out known indels included short insertions/deletions and consequently many, otherwise common indels in the dog species might have remained in the dataset after filtering. Furthermore, the number of previously published indels was also ∼40% less numerous than the number of SNPs published in the same study and ∼80% less than the number of SNP in the combined SNP databases. Misalignment of reads, e.g., from different copy-number variant regions (including genes of the same gene family) might also have contributed to the higher number of shared indels. Such errors could have also occurred in the reference sequence, resulting in the detection of false positive indels. Finally, false positive indels could be also technical artifacts from the sequencing. As a result of these possible explanations, biologically relevant indels cannot be distinguished from false positives and further studies will be required for proper interpretation of these findings.

We examined the 12 genes with START and STOP codon mutations in more detail. First of all, all of the *loss of function* SNP mutations were in a heterozygous form, i.e., both of the individuals carried at least 1 complete copy of the affected 12 genes. In the case of the ENSCAFG00000008061 gene, the average depth of coverage at the SNP position was significantly lower (by ∼70%) when compared to the average depth of coverage of the whole genome of the same individual. The depth of coverage was especially low for a ∼440 bp long segment surrounding the mutation, with a short segment completely missing. This is probably because this particular region contains repetitive sequences with a high GC content (77% on average). This segment also partially overlaps with the first exon of the gene. In addition, the sequence coverage pattern of this particular region was very similar in the old_rep2 individual as well, in which the mutation was not detected. Therefore, this particular SNP is most likely a false positive, probably due to an incorrectly annotated gene on the reference genome. Out of the remaining 11 genes, 2 genes were without a human homolog and both of them were categorized as novel genes in Ensembl (i.e., only protein and cDNA sequence alignment support is available for the gene; these genes are: ENSCAFG00000017946 and ENSCAFG00000030632); currently no information is available on their functions that could link them to longevity and/or aging. The remaining nine genes had homologs in humans. In three of these genes (ENSCAFG00000011630, ENSCAFG00000007858 and the already mentioned ENSCAFG00000008061), the close proximity (50–500 bp regions) of the disruptive mutations was not conserved between dogs and humans based on the reference genome sequences. Furthermore, two genes with a human homolog (the previously mentioned ENSCAFG00000011630 and the ENSCAFG00000032497 gene) had a relatively low cDNA sequence similarity between the reference genomes of the two species (40–60% as compared to 80–97% in case of the remaining seven genes). The remaining seven genes included six known (ENSCAFG00000000433, ENSCAFG00000002007, ENSCAFG00000004279, ENSCAFG00000007858, ENSCAFG00 000010498, ENSCAFG00000029250) and one novel (ENSC AFG00000024414) gene and are likely to be true positive hits. Some of the stop codon gain mutations were located at the beginning of the genes (the ENSCAFG00000029250, ENSCAFG00000024414 genes, where the 28^*th*^ and 390^*th*^ nucleotide positions were affected, out of 3390 and 1755 nucleotides, respectively) having most likely a major, inactivating effect, while in the ENSCAFG00000010498 gene the mutation was located near the 3′ end of the gene (at the 3116^*th*^ nucleotide out of 3261) and might not be influential regarding protein functionality. Finally, it is worth mentioning that the potential loss-of-function effect of these mutations might be alleviated, if the genes are present in multiple copies in the canine genome, although this is unlikely in case of the genes with missing segments. In case of multicopy-genes, 1 (or more) copy of the gene could be a pseudogene, although currently none of these genes are marked as a pseudogene in Ensembl.

In our dataset, only a small proportion of the exonic mutations located in the pre-defined set of age-related genes were missense (Table 4). In comparison, Han et al. (2013) found 710 non-synonymous mutations (previously known and novel SNPs combined) at the 988 investigated genes when studying six human centenarians, out of which 89 SNPs were novel. We identified 670 novel missense mutations in genes in total, 24 of which were located in the pre-defined age-related genes. The increased number of novel SNPs identified in this study is partly because our data was not limited to a pre-defined set of genes and partly because less data was available in the canine SNP databases as compared to the human databases used by Han et al. (2013) and therefore it is likely that fewer common SNPs were filtered out. On the other hand, the smaller number of novel SNPs identified at the pre-defined age-related gene set is probably due to the smaller sample size in our study. Although the genetic pathways linked to these genes were already associated with aging and longevity, only one of them, the cholinergic receptor nicotinic alpha 3 subunit (canine ID: ENSCAFG00000001710; human ID: ENSG00000080644) was recently associated with longevity in three studies (Joshi et al., 2017; Pilling et al., 2017; Timmers et al., 2019, after Deelen et al., 2019). Therefore, the remaining genes could be candidates for longevity in dogs and their association with longevity should be further investigated once more Methuselah dog samples become available.

Most of the genes with human homologs presumably linked to longevity did not include novel SNPs in the studied dogs. One of the most likely reasons to this observation is that aging and longevity are both extremely complex phenotypes, influenced by many genetic (e.g., additive genetic effects, dominance, epistasis, etc.) and environmental factors. This makes it difficult to identify single causative genetic factors behind extreme longevity, as the phenotype may result from entirely different settings of additive pro-longevity factors in each individual, especially in an inter-species comparison. Also, the current study lacks statistical power to identify significant associations between genetic and phenotypic variables. Finally, a third possible explanation could also be that mutations located in other genes may indirectly affect the function of longevity related genes by altering regulatory mechanisms. We identified several missense mutations in genes that had a function in translational processes (e.g., ENSCAFG00000005572 or ENSCAFG00000000104) and thus may affect the synthesis of proteins in general, including possible aging-related regulatory factors.

Based on the whole genome sequence data of 722 dogs (the NHGRI database; Plassais et al., 2019), we could conclude that the missense SNP mutations at protein domains were mainly located in SNP-poor regions. The 722 dogs had on average 2.5 SNPs within a window of ∼290 bp around the missense mutations (only those at protein domains were considered). In contrast, the 2 Methuselah dogs had an average of 13.6 SNPs within the same windows, 10.7 of which were categorized as novel. This claim is further supported by the complete database we created by merging three publicly available SNP databases (a total of 61,180,804 SNPs were included): within a 200 bp symmetric window around these missense SNPs located at protein domains, ∼71% of the windows contained 5 SNPs or less in the database and 87% of them had 10 SNPs or less within the same window. Therefore, it is likely that the SNPs we identified in the Methuselah dogs are mostly functional. These closely located SNPs also influenced the same protein domains. Two of these genes (ENSCAFG00000002366 and ENSCAFG00000009496) and two others with 1 single SNP at protein domains (ENSCAFG00000023076 and ENSCAFG00000003564) are directly related to (the regulation of) gene transcription or translation, based on which we can formulate a hypothesis that there is a link between gene regulation and longevity. This is in accordance with the literature, where a recent study investigated 1805 LAGs across 205 species and found that “LAGs that have orthologs across a high number of phyla were enriched in translational processes” (quote from Yanai et al., 2017). The remaining gene with more than 1 SNP mutation at a protein domain is related to immune response (ENSCAFG00000018626). Genes including more than 1 missense SNP mutation in either one of the two individuals revealed mostly the same pattern in SNP distribution with only a few exceptions.

472 genes included a total of 670 missense variants in either one or in both of the analyzed samples. Seven of these genes were part of the “Wnt signaling pathway,” while 6 genes from each of the following pathways were also among the 472: inflammation mediated by chemokine and cytokine signaling pathway, the nicotinic acetylcholine receptor signaling pathway and the cadherin signaling pathway. Five genes were from the Alzheimer disease-presenilin pathway. These pathways define an interesting direction for future longevity research in dogs and model animals alike. In addition to the named genetic pathways, the genes themselves (as well as their homologs in other species) are principal candidates for association studies aiming to identify linkage between genes and long life expectancy.

### Main Limitation of the Study

The primary limitation of this study is the small sample size: for this study, two dogs of extreme age were sequenced, analyzed and compared to a set of 850 dogs of a presumably averagely long lifespan.

Aging is a complex trait with many genes, regulatory regions, environmental factors, etc. involved. Consequently, it is possible that dogs with an averagely long life also carry mutations that have a positive impact on the length of their lifespan and it is the combined effect of all loci and environmental factors that results in an overall shorter lifespan. Therefore, when we removed the previously published SNPs from the data, we might also have removed relevant mutations and focused only on the most promising targets. This was done because with samples from only two extremely old dogs, we did not have the statistical power to find all genomic loci influencing longevity. However, in the future, when more data become available, the mutations with smaller effects can also be investigated.

Consequently, this study is preliminary regarding the aim to detect mutations, which could be actually relevant in the context of lifespan determination in dogs. However, the findings of such studies can open up new ideas and directions for future investigations with higher statistical power.

## Conclusion

Here we presented a preliminary study aiming to investigate the genetic background of longevity and aging in dogs. By analyzing all canine genes, we detected rare variants that might be linked to canine longevity and identified genes as potential LAGs. Based on their gene ontologies, these genes were related – among others – to gene transcription/translation and its regulation, to immune response and to the nervous system in general. Based on these preliminary results, we hypothesize a possible link between extreme longevity and the regulation of gene transcription/translation. In a recently published study, Yanai et al. (2017) independently came to the same conclusion. This hypothesis (namely: a crucial genetic requirement of extreme longevity lies within the *fine-tuning* (i.e., the superior calibration) of RNA (and thereof protein) production of an organism) is an interesting direction for future research aiming to better understand longevity.

We also identified several disrupted genes, albeit in heterozygous forms and therefore both examined individuals carried at least 1 intact copy of the affected 12 genes. The two Methuselah dogs sequenced for this study were unrelated mixed-breed individuals, suggesting that the mutations detected in both of them are relevant. Therefore, the shared SNPs we found in the two individuals (and the related genes) could be targets in future age-related research. This is further supported by the fact that none of these mutations were found in databases containing information from a total of 850 dogs with an average lifespan, representing a wide range of breeds as well as mixed-breed individuals from multiple countries.

Finally, it is worth mentioning that the younger dog analyzed in this study (aged 22) could be considered on the human scale as centenarian, while the older one (aged 27) would be a supercentenarian. A comparison of these two groups was infeasible in the current study due to the lack of statistical power, which would have led to unreliable results. However, once more DNA samples from dogs of extreme age become available, such a comparison would become possible and would aid us understanding the genetics of exceptionally extreme longevity.

## Data Availability Statement

The raw sequence files analyzed in this study is publicly available at the [American] National Center for Biotechnology Information’s (NCBI) Sequence Read Archive (SRA), under the bioproject ID PRJNA586720 (biosample IDs: SAMN13164793 and SAMN13164794).

## Ethics Statement

The animal study was reviewed and approved by the Government Office of Pest County (Hungary). Written informed consent was obtained from the owners for the participation of their animals in this study.

## Author Contributions

EK, BE, and SS designed the experiments. SS, BE, and KT performed the wet-lab experiments. DJ did the computational experiments and analysis. DJ and SS wrote the manuscript. All authors read and approved the manuscript.

## Conflict of Interest

The authors declare that the research was conducted in the absence of any commercial or financial relationships that could be construed as a potential conflict of interest.

## Footnotes

1 http://bigd.big.ac.cn/dogsdv2/

## References

[B1] Adams V. J. Evans K. M. Sampson J. Wood J. L. (2010). Methods and mortality results of a health survey of purebred dogs in the UK. *J. Small Anim. Pract.* 51 512–524. 10.1111/j.1748-5827.2010.00974.x 21029096

[B2] Andrews S. (2010). *FastQC: A Quality Control Tool for High Throughput Sequence Data.* Available online at: https://www.bioinformatics.babraham.ac.uk/projects/fastqc/ (Accessed October 6, 2011).

[B3] Bai B. Zhao W. M. Tang B. X. Wang Y. Q. Wang L. Zhang Z. (2015). DoGSD: the dog and wolf genome SNP database. *Nucleic Acids Res.* 43 D777–D783. 10.1093/nar/gku1174 25404132 PMC4383968

[B4] Broad Institute (2009). *Picard Toolkit.* Available online at: GitHub Repository – http://broadinstitute.github.io/picard/ (Accessed July 16, 2018).

[B5] Chapagain D. Range F. Huber L. Virányi Z. (2018). Cognitive aging in dogs. *Gerontology* 64 165–171. 10.1159/000481621 29065419 PMC5841136

[B6] Debrabant B. Soerensen M. Flachsbart F. Dato S. Mengel-From J. Stevnsner T. (2014). Human longevity and variation in DNA damage response and repair: study of the contribution of sub-processes using competitive gene-set analysis. *Eur. J. Hum. Genet.* 22 1131–1136. 10.1038/ejhg.2013.299 24518833 PMC4135411

[B7] Deelen J. Evans D. S. Arking D. E. Tesi N. Nygaard M. Liu X. (2019). A meta-analysis of genome-wide association studies identifies multiple longevity genes. *Nat Commun.* 10:3669 10.1038/s41467-019-11558-11552 PMC6694136 31413261

[B8] Deelen J. Uh H. W. Monajemi R. van Heemst D. Thijssen P. E. Böhringer S. (2013). Gene set analysis of GWAS data for human longevity highlights the relevance of the insulin/IGF-1 signaling and telomere maintenance pathways. *Age* 35 235–249. 10.1007/s11357-011-9340-3 22113349 PMC3543749

[B9] Flatt T. Partridge L. (2018). Horizons in the evolution of aging. *BMC Biol.* 16:93. 10.1186/s12915-018-0562-z 30124168 PMC6100731

[B10] Gems D. Riddle D. L. (2000). Genetic, behavioral and environmental determinants of male longevity in *Caenorhabditis elegans*. *Genetics* 154 1597–1610. 10747056 10.1093/genetics/154.4.1597 PMC1461011

[B11] Gilmore K. M. Greer K. A. (2015). Why is the dog an ideal model for aging research? *Exp. Gerontol.* 71 14–20. 10.1016/j.exger.2015.08.008 26325590

[B12] Han J. Ryu S. Moskowitz D. M. Rothenberg D. Leahy D. J. Atzmon G. (2013). Discovery of novel non-synonymous SNP variants in 988 candidate genes from 6 centenarians by target capture and next-generation sequencing. *Mech. Ageing Dev.* 134 478–485. 10.1016/j.mad.2013.01.005 23376243 PMC3787996

[B13] Herskind A. M. McGue M. Holm N. V. Sørensen T. I. Harvald B. Vaupel J. W. (1996). The heritability of human longevity: a population-based study of 2872 Danish twin pairs born 1870-1900. *Hum. Genet.* 97 319–323. 10.1007/s004390050042 8786073

[B14] Hoffman J. M. Creevy K. E. Franks A. O’Neill D. G. Promislow D. E. L. (2018). The companion dog as a model for human aging and mortality. *Aging Cell* 17:e12737. 10.1111/acel.12737 29457329 PMC5946068

[B15] Huffman D. M. Deelen J. Ye K. Bergman A. Slagboom E. P. Barzilai N. (2012). Distinguishing between longevity and buffered-deleterious genotypes for exceptional human longevity: the case of the MTP gene. *J. Gerontol. A Biol. Sci. Med. Sci.* 67 1153–1160. 10.1093/gerona/gls103 22496539 PMC3668387

[B16] Hunt S. E. McLaren W. Gil L. Thormann A. Schuilenburg H. Sheppard D. (2018). Ensembl variation resources. *Database* 2018:bay119. 10.1093/database/bay119 30576484 PMC6310513

[B17] Inoue M. Kwan N. C. L. Sugiura K. (2018). Estimating the life expectancy of companion dogs in Japan using pet cemetery data. *J. Vet. Med. Sci.* 80 1153–1158. 10.1292/jvms.17-0384 29798968 PMC6068313

[B18] Jaul E. Barron J. (2017). Age-related diseases and clinical and public health implications for 85 years old and over population. *Front. Public Health* 11:335. 10.3389/fpubh.2017.00335 29312916 PMC5732407

[B19] Jimenez A. G. (2016). Physiological underpinnings in life-history trade-offs in man’s most popular selection experiment: the dog. *J. Comp. Physiol. B* 186 813–827. 10.1007/s00360-016-1002-4 27222254

[B20] Joshi P. K. Pirastu N. Kentistou K. A. Fischer K. Hofer E. Schraut K. E. (2017). Genome-wide meta-analysis associates HLA-DQA1/DRB1 and LPA and lifestyle factors with human longevity. *Nat. Commun.* 8:910. 10.1038/s41467-017-00934-5 29030599 PMC5715013

[B21] Kaeberlein M. Creevy K. E. Promislow D. E. L. (2016). The dog aging project: translational geroscience in companion animals. *Mamm. Genome* 27 279–288. 10.1007/s00335-016-9638-7 27143112 PMC4936929

[B22] Lehtovaara A. Schielzeth H. Flis I. Friberg U. (2013). Heritability of life span is largely sex limited in *Drosophila*. *Am. Nat.* 182 653–665. 10.1086/673296 24107372

[B23] Leroy G. Phocas F. Hedan B. Verrier E. Rognon X. (2015). Inbreeding impact on litter size and survival in selected canine breeds. *Vet. J.* 203 74–78. 10.1016/j.tvjl.2014.11.008 25475165

[B24] Leroy G. Verrier E. Merieaux J. C. Rognon X. (2009). Genetic diversity of dog breeds: within-breed diversity comparing genealogical and molecular data. *Anim. Genet.* 40 323–332. 10.1111/j.1365-2052.2008.01842.x 19222437

[B25] Li H. (2011). A statistical framework for SNP calling, mutation discovery, association mapping and population genetical parameter estimation from sequencing data. *Bioinformatics* 27 2987–2993. 10.1093/bioinformatics/btr509 21903627 PMC3198575

[B26] Li H. Durbin R. (2009). Fast and accurate short read alignment with Burrows-Wheeler Transform. *Bioinformatics* 25 1754–1760. 10.1093/bioinformatics/btp324 19451168 PMC2705234

[B27] Lindblad-Toh K. Garber M. Zuk O. Lin M. F. Parker B. J. Washietl S. (2011). A high-resolution map of human evolutionary constraint using 29 mammals. *Nature* 478 476–482. 10.1038/nature10530 21993624 PMC3207357

[B28] Lindblad-Toh K. Wade C. M. Mikkelsen T. S. Karlsson E. K. Jaffe D. B. Kamal M. (2005). Genome sequence, comparative analysis and haplotype structure of the domestic dog. *Nature* 438 803–819. 16341006 10.1038/nature04338

[B29] López-Otín C. Blasco M. A. Partridge L. Serrano M. Kroemer G. (2013). The hallmarks of aging. *Cell* 153 1194–1217. 10.1016/j.cell.2013.05.039 23746838 PMC3836174

[B30] McLaren W. Gil L. Hunt S. E. Riat H. S. Ritchie G. R. Thormann A. (2016). The ensembl variant effect predictor. *Genome Biol.* 17:122. 10.1186/s13059-016-0974-4 27268795 PMC4893825

[B31] Moskalev A. A. Shaposhnikov M. V. Plyusnina E. N. Zhavoronkov A. Budovsky A. Yanai H. (2013). The role of DNA damage and repair in aging through the prism of Koch-like criteria. *Ageing Res. Rev.* 12 661–684. 10.1016/j.arr.2012.02.001 22353384

[B32] Ostrander E. A. Wayne R. K. Freedman A. H. Davis B. W. (2017). Demographic history, selection and functional diversity of the canine genome. *Nat. Rev. Genet.* 18 705–720. 10.1038/nrg.2017.67 28944780

[B33] Pilling L. C. Kuo C. L. Sicinski K. Tamosauskaite J. Kuchel G. A. Harries L. W. (2017). Human longevity: 25 genetic loci associated in 389,166 UK biobank participants. *Aging* 9 2504–2520. 10.18632/aging.101334 29227965 PMC5764389

[B34] Piper M. D. W. Selman C. McElwee J. J. Partridge L. (2008). Separating cause from effect: how does insulin/IGF signalling control lifespan in worms, flies and mice? *J. Intern. Med.* 263 179–191. 10.1111/j.1365-2796.2007.01906.x 18226095

[B35] Plassais J. Kim J. Davis B. W. Karyadi D. M. Hogan A. N. Harris A. C. (2019). Whole genome sequencing of canids reveals genomic regions under selection and variants influencing morphology. *Nat. Commun.* 10:1489. 10.1038/s41467-019-09373-w 30940804 PMC6445083

[B36] Ruby J. G. Wright K. M. Rand K. A. Kermany A. Noto K. Curtis D. (2018). Estimates of the heritability of human longevity are substantially inflated due to assortative mating. *Genetics* 210 1109–1124. 10.1534/genetics.118.301613 30401766 PMC6218226

[B37] Sanders A. E. Wang C. Katz M. Derby C. A. Barzilai N. Ozelius L. (2010). Association of a functional polymorphism in the cholesteryl ester transfer protein (CETP) gene with memory decline and incidence of dementia. *JAMA* 303 150–158. 10.1001/jama.2009.1988 20068209 PMC3047443

[B38] Sándor S. Kubinyi E. (2019). Genetic pathways of aging and their relevance in the dog as a natural model of human cognitive aging. *Front. Genet.* 10:948. 10.3389/fgene.2019.00948 31681409 PMC6813227

[B39] Sebastiani P. Nussbaum L. Andersen S. L. Black M. J. Perls T. T. (2016). Increasing sibling relative risk of survival to older and older ages and the importance of precise definitions of “Aging,” “Life Span,” and “Longevity”. *J. Gerontol. A Biol. Sci. Med. Sci.* 71 340–346. 10.1093/gerona/glv020 25814633 PMC4757962

[B40] Sims D. Sudbery I. Ilott N. E. Heger A. Ponting C. P. (2014). Sequencing depth and coverage: key considerations in genomic analyses. *Nat. Rev. Genet.* 15 121–132. 10.1038/nrg3642 24434847

[B41] Timmers P. R. Mounier N. Lall K. Fischer K. Ning Z. Feng X. (2019). Genomics of 1 million parent lifespans implicates novel pathways and common diseases and distinguishes survival chances. *eLife* 8:e39856. 10.7554/eLife.39856 30642433 PMC6333444

[B42] United Nations (2015). *Department of Economic, and Social Affairs, Population Division 2015.* New York, NY: United Nations.

[B43] Van der Auwera G. A. Carneiro M. Hartl C. Poplin R. del Angel G. Levy-Moonshine A. (2013). From FastQ data to high-confidence variant calls: the genome analysis toolkit best practices pipeline. *Curr. Protoc. Bioinform.* 43:11. 10.1002/0471250953.bi1110s43 25431634 PMC4243306

[B44] Wallis L. J. Szabó D. Erdélyi-Belle B. Kubinyi E. (2018). Demographic change across the lifespan of pet dogs and their impact on health status. *Front. Vet. Sci.* 5:200 10.3389/fvets.2018.00200 PMC6115627 30191153

[B45] World Health Organization [WHO] (2018). *World Health Statistics 2018: Monitoring Health for the SDGs, Sustainable Development Goals.* Geneva: World Health Organization.

[B46] Yanai H. Budovsky A. Barzilay T. Tacutu R. Fraifeld V. E. (2017). Wide-scale comparative analysis of longevity genes and interventions. *Aging Cell* 16 1267–1275. 10.1111/acel.12659 28836369 PMC5676071

[B47] Yanai H. Fraifeld V. E. (2018). The role of cellular senescence in aging through the prism of Koch-like criteria. *Ageing Res. Rev.* 41 18–33. 10.1016/j.arr.2017.10.004 29106993

