# Supplementary material for: A Preliminary Study to Investigate the Genetic Background of Longevity Based on Whole-Genome Sequence Data of Two Methuselah Dogs
Source: Front Genet. 2020 Apr 16;11:315. doi: 10.3389/fgene.2020.00315 (PMC7176982; doi:10.3389/fgene.2020.00315)
Supplement: Supplementary file 1 [file Data_Sheet_1.docx]

| 1. Number of SNP for different annotation categories. | | | |
| --- | --- | --- | --- |
| **Annotation category** | **old rep1** | **old rep2** | **Overlap** |
| 3 prime UTR variant | 338 | 554 | 38 |
| 5 prime UTR variant | 436 | 539 | 155 |
| coding sequence variant | 1 | 0 | 0 |
| downstream gene variant | 3526 | 4712 | 824 |
| intron variant | 48806 | 55628 | 8361 |
| intron variant, non coding transcript variant | 4433 | 4572 | 639 |
| mature miRNA variant | 1 | 3 | 1 |
| missense variant | 918 | 876 | 109 |
| missense variant, splice region variant | 10 | 11 | 1 |
| non coding transcript exon variant | 346 | 359 | 81 |
| splice acceptor variant | 8 | 5 | 0 |
| splice donor variant | 5 | 3 | 3 |
| splice donor variant, non coding transcript variant | 0 | 1 | 0 |
| splice region variant, intron variant | 75 | 94 | 27 |
| splice region variant, intron variant, non coding transcript variant | 1 | 4 | 0 |
| splice region variant, non coding transcript exon variant | 0 | 1 | 0 |
| splice region variant, synonymous variant | 15 | 17 | 3 |
| start lost | 1 | 6 | 0 |
| stop gained | 10 | 12 | 0 |
| stop lost | 1 | 0 | 0 |
| synonymous variant | 856 | 826 | 152 |
| upstream gene variant | 4778 | 6098 | 1520 |

| 1. List of genes with non-synonymous exon mutations in the two methuselah dogs. The 19 age-related genes are also highlighted. | | | | |
| --- | --- | --- | --- | --- |
| **Gene ID** | **Age-related gene** | **old_rep1** | **old_rep2** | **GO terms** |
| ENSCAFG00000014403 | + | + | + | GO:0016020, GO:0016021 |
| ENSCAFG00000002182 | + | + | - | GO:0000166, GO:0004365, GO:0005829, GO:0006006, GO:0006096, GO:0016491, GO:0016620, GO:0050661, GO:0051287, GO:0055114 |
| ENSCAFG00000004568 | + | + | - | GO:0001701, GO:0001966, GO:0005509, GO:0005737, GO:0005768, GO:0005783, GO:0005794, GO:0005798, GO:0005903, GO:0005905, GO:0006898, GO:0006972, GO:0007584, GO:0008144, GO:0008328, GO:0008344, GO:0009617, GO:0015889, GO:0016247, GO:0016324, GO:0020028, GO:0030135, GO:0030139, GO:0030492, GO:0030666, GO:0031526, GO:0032589, GO:0038023, GO:0038024, GO:0040012, GO:0042366, GO:0042803, GO:0042953, GO:0043202, GO:0045177, GO:0045202, GO:0046331, GO:0051968, GO:0070062, GO:0070207 |
| ENSCAFG00000013332 | + | + | - | GO:0000977, GO:0000980, GO:0001077, GO:0001102, GO:0001158, GO:0001228, GO:0003151, GO:0003676, GO:0003677, GO:0003682, GO:0003700, GO:0004402, GO:0005634, GO:0005654, GO:0005737, GO:0005741, GO:0006355, GO:0006357, GO:0006366, GO:0006970, GO:0006974, GO:0008140, GO:0010628, GO:0016020, GO:0016525, GO:0016573, GO:0019901, GO:0031573, GO:0032915, GO:0035497, GO:0035861, GO:0043565, GO:0045444, GO:0045944, GO:0051091, GO:0060612, GO:0110024, GO:1902110 |
| ENSCAFG00000024527 | + | + | - | GO:0004888, GO:0005216, GO:0005230, GO:0005887, GO:0006811, GO:0007165, GO:0007204, GO:0007268, GO:0016020, GO:0016021, GO:0034220, GO:0042391, GO:0043005, GO:0045202, GO:0050877 |
| ENSCAFG00000001710 | + | + | - | GO:0004888, GO:0005216, GO:0005230, GO:0005886, GO:0005887, GO:0005892, GO:0006811, GO:0007165, GO:0007268, GO:0007271, GO:0007274, GO:0007399, GO:0015464, GO:0016020, GO:0016021, GO:0022848, GO:0030054, GO:0034220, GO:0035094, GO:0035095, GO:0042391, GO:0043005, GO:0045202, GO:0045211, GO:0050877, GO:0060078, GO:0060079, GO:0060084 |
| ENSCAFG00000001906 | + | + | - | GO:0001934, GO:0003149, GO:0003150, GO:0003151, GO:0004888, GO:0005102, GO:0005109, GO:0005886, GO:0007166, GO:0007267, GO:0007275, GO:0009986, GO:0016020, GO:0016021, GO:0016055, GO:0017147, GO:0030165, GO:0030514, GO:0030855, GO:0035425, GO:0035567, GO:0042493, GO:0042813, GO:0044338, GO:0044339, GO:0045892, GO:0045893, GO:0051091, GO:0060022, GO:0060070, GO:0060412, GO:0090090, GO:0090179, GO:1903204 |
| ENSCAFG00000008746 | + | + | - | GO:0004435, GO:0005509, GO:0005622, GO:0005737, GO:0005829, GO:0006629, GO:0007165, GO:0008081, GO:0016020, GO:0016042, GO:0016787, GO:0035556, GO:0043231, GO:0048015, GO:0050429 |
| ENSCAFG00000006713 | + | - | + | GO:0005886, GO:0008201, GO:0016020, GO:0016021, GO:0031694, GO:0031695, GO:0031696, GO:0042802, GO:0046914, GO:0048471, GO:0071874, GO:0106072 |
| ENSCAFG00000010840 | + | - | + | GO:0001819, GO:0004930, GO:0004935, GO:0004938, GO:0005737, GO:0005887, GO:0006940, GO:0007165, GO:0007186, GO:0007188, GO:0016020, GO:0016021, GO:0019229, GO:0019901, GO:0030168, GO:0030335, GO:0031696, GO:0031996, GO:0032147, GO:0032148, GO:0032795, GO:0032870, GO:0035624, GO:0042593, GO:0042803, GO:0043235, GO:0043406, GO:0043410, GO:0045741, GO:0046676, GO:0046982, GO:0050995, GO:0051044, GO:0051379, GO:0051380, GO:0061179, GO:0071875, GO:0071880, GO:0071881, GO:0071882, GO:0090303 |
| ENSCAFG00000011630 | + | - | + | GO:0016020, GO:0016021 |
| ENSCAFG00000023904 | + | - | + | GO:0001508, GO:0004888, GO:0005216, GO:0005230, GO:0005886, GO:0005887, GO:0005892, GO:0006811, GO:0006939, GO:0006940, GO:0007165, GO:0007268, GO:0007271, GO:0007274, GO:0007626, GO:0015464, GO:0016020, GO:0016021, GO:0022848, GO:0030054, GO:0034220, GO:0035094, GO:0035095, GO:0042391, GO:0043005, GO:0045202, GO:0045211, GO:0050877, GO:0051971, GO:0060078, GO:0060079, GO:0060084 |
| ENSCAFG00000025598 | + | - | + | GO:0000077, GO:0000724, GO:0001932, GO:0005634, GO:0005730, GO:0006289, GO:0006468, GO:0006974, GO:0007093, GO:0008156, GO:0009411, GO:0009792, GO:0030896, GO:0031573, GO:0033314, GO:0035861, GO:0044778, GO:0071479 |
| ENSCAFG00000001844 | + | - | + | GO:0051087 |
| ENSCAFG00000007985 | + | - | + | GO:0000187, GO:0004435, GO:0004629, GO:0005085, GO:0005622, GO:0005829, GO:0005886, GO:0006629, GO:0007165, GO:0007200, GO:0007264, GO:0007265, GO:0008081, GO:0008277, GO:0016042, GO:0016787, GO:0017016, GO:0019899, GO:0032835, GO:0035556, GO:0045859, GO:0046578 |
| ENSCAFG00000013670 | + | - | + | GO:0000226, GO:0005737, GO:0005829, GO:0005856, GO:0005874, GO:0005886, GO:0007613, GO:0008017, GO:0010469, GO:0010506, GO:0010629, GO:0010917, GO:0015630, GO:0015631, GO:0016020, GO:0016607, GO:0017124, GO:0019899, GO:0019901, GO:0030424, GO:0030425, GO:0030426, GO:0030673, GO:0031110, GO:0031116, GO:0031175, GO:0032930, GO:0033673, GO:0034185, GO:0034399, GO:0036464, GO:0036477, GO:0042802, GO:0042803, GO:0043005, GO:0043025, GO:0044297, GO:0045298, GO:0045773, GO:0048018, GO:0048312, GO:0050808, GO:0050848, GO:0051087, GO:0051879, GO:0070507, GO:0071813, GO:0090258, GO:0097418, GO:0097435, GO:0099609, GO:1900034, GO:1901216, GO:1902474, GO:1903748, GO:1903829, GO:1904428, GO:1990000, GO:2001020 |
| ENSCAFG00000015046 | + | - | + | GO:0000045, GO:0000423, GO:0001932, GO:0001933, GO:0001934, GO:0005776, GO:0005930, GO:0006914, GO:0008333, GO:0009267, GO:0010608, GO:0016236, GO:0016240, GO:0034045, GO:0035032, GO:0042149, GO:0043552, GO:0044233, GO:0045335, GO:0051020, GO:0090207, GO:0097629, GO:0097632, GO:0098780 |
| ENSCAFG00000015914 | + | - | + | GO:0005634, GO:0005730, GO:0071391 |
| ENSCAFG00000018622 | + | - | + | GO:0001650, GO:0001934, GO:0005886, GO:0005905, GO:0006898, GO:0008022, GO:0010718, GO:0010862, GO:0030136, GO:0030335, GO:0030511, GO:0032091, GO:0032436, GO:0035026, GO:0035615, GO:0038024, GO:0043066, GO:0043231, GO:0045807, GO:0045892, GO:0045893, GO:0046332, GO:0060391, GO:0060766, GO:0070022, GO:0090090, GO:1903077, GO:2000096, GO:2000370, GO:2000643 |
| ENSCAFG00000002184 | - | + | + | GO:0005198, GO:0005882, GO:0045095 |
| ENSCAFG00000002366 | - | + | + | GO:0000028, GO:0003723, GO:0003729, GO:0003735, GO:0005840, GO:0006412, GO:0006450, GO:0015935, GO:0019843, GO:0022627, GO:1990904 |
| ENSCAFG00000003564 | - | + | + | GO:0000027, GO:0003735, GO:0005622, GO:0005840, GO:0006412 |
| ENSCAFG00000004690 | - | + | + | GO:0000166, GO:0003676, GO:0003743, GO:0004004, GO:0004386, GO:0005524, GO:0006413, GO:0016787 |
| ENSCAFG00000008253 | - | + | + | GO:0000166, GO:0005524 |
| ENSCAFG00000009072 | - | + | + | GO:0005216, GO:0005244, GO:0005249, GO:0005251, GO:0005267, GO:0006811, GO:0006813, GO:0008076, GO:0016020, GO:0016021, GO:0019894, GO:0030424, GO:0030673, GO:0032590, GO:0032809, GO:0034765, GO:0043025, GO:0044325, GO:0051260, GO:0051262, GO:0055085, GO:0071805 |
| ENSCAFG00000009496 | - | + | + | GO:0001958, GO:0005634, GO:0006355, GO:0008134, GO:0014037, GO:0042552, GO:0045682, GO:0045892 |
| ENSCAFG00000012846 | - | + | + | GO:0004672, GO:0004674, GO:0005524, GO:0005634, GO:0005737, GO:0005829, GO:0006468, GO:0035091, GO:0035556, GO:0043576, GO:0045719, GO:0046777, GO:0070092, GO:0097009 |
| ENSCAFG00000014734 | - | + | + | GO:0005262, GO:0016020, GO:0016021, GO:0034704, GO:0050982, GO:0070588, GO:0097730 |
| ENSCAFG00000015116 | - | + | + | GO:0000166, GO:0005524 |
| ENSCAFG00000016031 | - | + | + | GO:0000166, GO:0000795, GO:0001673, GO:0001934, GO:0005524, GO:0005634, GO:0005737, GO:0005829, GO:0005886, GO:0006986, GO:0007140, GO:0007141, GO:0007283, GO:0007286, GO:0009408, GO:0009409, GO:0009986, GO:0010971, GO:0016192, GO:0016887, GO:0019899, GO:0031072, GO:0032781, GO:0034605, GO:0034620, GO:0036128, GO:0042026, GO:0042623, GO:0043209, GO:0044183, GO:0051082, GO:0051085, GO:0051787, GO:0051861, GO:0070194, GO:0072687, GO:0090084, GO:0097718, GO:1901896 |
| ENSCAFG00000018626 | - | + | + | GO:0001906, GO:0005579, GO:0005615, GO:0005829, GO:0005886, GO:0006955, GO:0051260 |
| ENSCAFG00000019059 | - | + | + | GO:0005737, GO:0005765, GO:0005829, GO:0016236, GO:0019898, GO:0031901, GO:0031902, GO:0032510, GO:0032991, GO:0036503 |
| ENSCAFG00000023076 | - | + | + | GO:0000977, GO:0003676, GO:0003677, GO:0005634, GO:0008168, GO:0016740, GO:0019233, GO:0022008, GO:0031175, GO:0031490, GO:0032259, GO:0043565, GO:0046872, GO:0050965, GO:0051574, GO:1900111, GO:1990226 |
| ENSCAFG00000000361 | - | + | + | GO:0005096, GO:0005829, GO:0005886, GO:0005929, GO:0006886, GO:0017137, GO:0036064, GO:0043547, GO:0090630, GO:1902018 |
| ENSCAFG00000000945 | - | + | + | GO:0000139, GO:0003824, GO:0004571, GO:0005509, GO:0005783, GO:0005793, GO:0005794, GO:0005829, GO:0006491, GO:0008152, GO:0016020, GO:0016787, GO:0016798 |
| ENSCAFG00000010345 | - | + | + | GO:0002089, GO:0005886, GO:0005911, GO:0005913, GO:0007155, GO:0007156, GO:0007157, GO:0007286, GO:0009566, GO:0016020, GO:0016021, GO:0038023, GO:0042803, GO:0043296, GO:0044291, GO:0050839, GO:0060042, GO:0061951, GO:0098609, GO:1902414 |
| ENSCAFG00000010719 | - | + | + | GO:0001755, GO:0005615, GO:0005887, GO:0030215, GO:0030335, GO:0038191, GO:0045499, GO:0048843, GO:0050919, GO:0071526 |
| ENSCAFG00000010803 | - | + | + | GO:0005634, GO:0005737, GO:0005794, GO:0005813, GO:0005815, GO:0019899, GO:0030016, GO:0032947, GO:0034622, GO:1903358 |
| ENSCAFG00000012522 | - | + | + | GO:0000166, GO:0004017, GO:0005524, GO:0005525, GO:0005739, GO:0005759, GO:0006139, GO:0006172, GO:0016301, GO:0016310, GO:0016740, GO:0016776, GO:0019205, GO:0046033, GO:0046039, GO:0046041, GO:0046899, GO:0046940 |
| ENSCAFG00000012991 | - | + | + | GO:0000122, GO:0000977, GO:0001227, GO:0001822, GO:0003677, GO:0003713, GO:0005634, GO:0006355, GO:0006807, GO:0008584, GO:0008585, GO:0009791, GO:0010761, GO:0030325, GO:0035264, GO:0044212, GO:0045444, GO:0048008, GO:0048468, GO:0048644, GO:0048705, GO:0051091, GO:0060021, GO:0060325, GO:0060612, GO:0060613, GO:1903508, GO:1990830 |
| ENSCAFG00000015266 | - | + | + | GO:0003677, GO:0003714, GO:0005634, GO:0005730, GO:0005737, GO:0006355, GO:0008134, GO:0022904, GO:0032922, GO:0042149, GO:0042564, GO:0043231, GO:0043565, GO:0045892, GO:0070888, GO:0071158, GO:0072332, GO:2000210 |
| ENSCAFG00000015835 | - | + | + | GO:0000122, GO:0000228, GO:0000381, GO:0000776, GO:0000978, GO:0000993, GO:0001097, GO:0003677, GO:0003682, GO:0003690, GO:0003697, GO:0003714, GO:0003723, GO:0003725, GO:0003727, GO:0003730, GO:0003779, GO:0005524, GO:0005634, GO:0005654, GO:0005697, GO:0005813, GO:0007346, GO:0008143, GO:0009048, GO:0009986, GO:0016363, GO:0016607, GO:0017069, GO:0017130, GO:0030496, GO:0031490, GO:0032211, GO:0032922, GO:0032991, GO:0033673, GO:0034046, GO:0034244, GO:0036002, GO:0036464, GO:0042802, GO:0043021, GO:0043565, GO:0044877, GO:0045944, GO:0048255, GO:0051457, GO:0055013, GO:0070034, GO:0070934, GO:0070937, GO:0071013, GO:0071385, GO:0072686, GO:0090336, GO:0090575, GO:0098577, GO:0099122, GO:1901673, GO:1902275, GO:1902425, GO:1902889, GO:1990023, GO:1990280, GO:1990498, GO:1990830, GO:1990837, GO:1990841, GO:1990845, GO:1990904, GO:2000648, GO:2000737 |
| ENSCAFG00000016070 | - | + | + | GO:0006629, GO:0007216, GO:0007405, GO:0016020, GO:0016021, GO:0016787, GO:0019369, GO:0022008, GO:0042136, GO:0043196, GO:0045211, GO:0046340, GO:0071926, GO:0098921 |
| ENSCAFG00000016758 | - | + | + | GO:0000790, GO:0003676, GO:0003723, GO:0005634, GO:0008013, GO:0008134, GO:0008168, GO:0016607, GO:0016740, GO:0018024, GO:0032259, GO:0035097, GO:0042800, GO:0048188, GO:1902036, GO:1902275 |
| ENSCAFG00000022730 | - | + | + | GO:0004129, GO:0005739, GO:0005751, GO:0006123, GO:0008535, GO:0009055, GO:0009060, GO:0015002, GO:0015453, GO:0016020, GO:0016021, GO:0019646, GO:0022904, GO:0045277, GO:0055085, GO:1902600 |
| ENSCAFG00000022739 | - | + | + | GO:0003954, GO:0005739, GO:0005743, GO:0005747, GO:0008137, GO:0016020, GO:0016021, GO:0016491, GO:0042773, GO:0055114, GO:0070469 |
| ENSCAFG00000023848 | - | + | + | GO:0000122, GO:0000978, GO:0001078, GO:0003677, GO:0003700, GO:0005634, GO:0006357, GO:0042803 |
| ENSCAFG00000029208 | - | + | + |  |
| ENSCAFG00000029497 | - | + | + |  |
| ENSCAFG00000029689 | - | + | + | GO:0003824 |
| ENSCAFG00000030210 | - | + | + | GO:0016607 |
| ENSCAFG00000030254 | - | + | + |  |
| ENSCAFG00000030487 | - | + | + | GO:0016020, GO:0016021 |
| ENSCAFG00000030582 | - | + | + |  |
| ENSCAFG00000030864 | - | + | + |  |
| ENSCAFG00000031089 | - | + | + |  |
| ENSCAFG00000031444 | - | + | + | GO:0045095 |
| ENSCAFG00000031565 | - | + | + |  |
| ENSCAFG00000031685 | - | + | + |  |
| ENSCAFG00000031808 | - | + | + |  |
| ENSCAFG00000031834 | - | + | + |  |
| ENSCAFG00000032525 | - | + | + | GO:0005634, GO:0005737, GO:0005829, GO:0005886, GO:0015629, GO:0030315, GO:0031982, GO:0042383, GO:0043034, GO:0043484, GO:0044291, GO:0044548, GO:0045121, GO:0051259, GO:0070062, GO:0097493, GO:1901385 |
| ENSCAFG00000032561 | - | + | + |  |
| ENSCAFG00000032607 | - | + | + |  |

| 1. List of genes with non-synonymous exon mutations in protein domains in the 2 analyzed dogs. The human homologs of the genes are also provided when available. | | | |
| --- | --- | --- | --- |
| **Genelist** | **old_rep1** | **old_rep2** | **Human homolog** |
| ENSCAFG00000002184 | + | + | ENSG00000170421 |
| ENSCAFG00000002366 | + | + | ENSG00000083845 |
| ENSCAFG00000003564 | + | + | ENSG00000100316 |
| ENSCAFG00000004690 | + | + |  |
| ENSCAFG00000008253 | + | + |  |
| ENSCAFG00000009072 | + | + | ENSG00000129159 |
| ENSCAFG00000009496 | + | + | ENSG00000138386 |
| ENSCAFG00000014734 | + | + | ENSG00000158683 |
| ENSCAFG00000015116 | + | + |  |
| ENSCAFG00000016031 | + | + | ENSG00000126803 |
| ENSCAFG00000018626 | + | + | ENSG00000113600 |
| ENSCAFG00000019059 | + | + | ENSG00000167671 |
| ENSCAFG00000023076 | + | + | ENSG00000130711 |
| ENSCAFG00000000246 | + | - | ENSG00000050767 |
| ENSCAFG00000000433 | + | - | ENSG00000131459 |
| ENSCAFG00000000482 | + | - | ENSG00000037280 |
| ENSCAFG00000001161 | + | - | ENSG00000154358 |
| ENSCAFG00000001264 | + | - | ENSG00000171045 |
| ENSCAFG00000002106 | + | - | ENSG00000167721 |
| ENSCAFG00000002169 | + | - | ENSG00000165030 |
| ENSCAFG00000002182 | + | - | ENSG00000111640 |
| ENSCAFG00000002928 | + | - | ENSG00000165124 |
| ENSCAFG00000003165 | + | - | ENSG00000172331 |
| ENSCAFG00000003752 | + | - |  |
| ENSCAFG00000003962 | + | - | ENSG00000196159 |
| ENSCAFG00000004219 | + | - | ENSG00000165312 |
| ENSCAFG00000004229 | + | - | ENSG00000182890 |
| ENSCAFG00000004568 | + | - | ENSG00000107611 |
| ENSCAFG00000004830 | + | - | ENSG00000033050 |
| ENSCAFG00000004958 | + | - | ENSG00000285505 |
| ENSCAFG00000005132 | + | - | ENSG00000070526 |
| ENSCAFG00000005446 | + | - |  |
| ENSCAFG00000005530 | + | - | ENSG00000128016 |
| ENSCAFG00000005725 | + | - | ENSG00000157637 |
| ENSCAFG00000006221 | + | - |  |
| ENSCAFG00000006421 | + | - | ENSG00000183087 |
| ENSCAFG00000006520 | + | - | ENSG00000171126 |
| ENSCAFG00000007240 | + | - | ENSG00000086200 |
| ENSCAFG00000007241 | + | - | ENSG00000166347 |
| ENSCAFG00000007504 | + | - | ENSG00000101444 |
| ENSCAFG00000007892 | + | - | ENSG00000156575 |
| ENSCAFG00000008040 | + | - |  |
| ENSCAFG00000008719 | + | - | ENSG00000109756 |
| ENSCAFG00000009001 | + | - | ENSG00000147459 |
| ENSCAFG00000009273 | + | - | ENSG00000010327 |
| ENSCAFG00000009509 | + | - | ENSG00000124491 |
| ENSCAFG00000009617 | + | - | ENSG00000185745 |
| ENSCAFG00000009720 | + | - | ENSG00000273749 |
| ENSCAFG00000010406 | + | - | ENSG00000139117 |
| ENSCAFG00000010846 | + | - | ENSG00000141959 |
| ENSCAFG00000011109 | + | - | ENSG00000167792 |
| ENSCAFG00000011348 | + | - | ENSG00000169129 |
| ENSCAFG00000011504 | + | - | ENSG00000150337 |
| ENSCAFG00000011687 | + | - | ENSG00000172932 |
| ENSCAFG00000012005 | + | - | ENSG00000119973 |
| ENSCAFG00000012530 | + | - | ENSG00000152104 |
| ENSCAFG00000012650 | + | - | ENSG00000172425 |
| ENSCAFG00000012846 | + | - | ENSG00000115687 |
| ENSCAFG00000013143 | + | - | ENSG00000160613 |
| ENSCAFG00000013202 | + | - | ENSG00000149577 |
| ENSCAFG00000013275 | + | - | ENSG00000075711 |
| ENSCAFG00000013332 | + | - | ENSG00000115966 |
| ENSCAFG00000013617 | + | - | ENSG00000138413 |
| ENSCAFG00000014030 | + | - | ENSG00000011376 |
| ENSCAFG00000014033 | + | - | ENSG00000180817 |
| ENSCAFG00000014195 | + | - | ENSG00000283154 |
| ENSCAFG00000014465 | + | - | ENSG00000182450 |
| ENSCAFG00000014730 | + | - | ENSG00000087269 |
| ENSCAFG00000015699 | + | - | ENSG00000138134 |
| ENSCAFG00000016229 | + | - | ENSG00000215193 |
| ENSCAFG00000016828 | + | - | ENSG00000005243 |
| ENSCAFG00000017712 | + | - | ENSG00000140749 |
| ENSCAFG00000017946 | + | - |  |
| ENSCAFG00000018376 | + | - | ENSG00000099814 |
| ENSCAFG00000018448 | + | - | ENSG00000211891 |
| ENSCAFG00000019108 | + | - |  |
| ENSCAFG00000019380 | + | - | ENSG00000148408 |
| ENSCAFG00000019416 | + | - | ENSG00000157881 |
| ENSCAFG00000019685 | + | - | ENSG00000187796 |
| ENSCAFG00000019848 | + | - | ENSG00000130635 |
| ENSCAFG00000023508 | + | - | ENSG00000013288 |
| ENSCAFG00000023635 | + | - | ENSG00000196266 |
| ENSCAFG00000023636 | + | - | ENSG00000005001 |
| ENSCAFG00000023735 | + | - | ENSG00000158477 |
| ENSCAFG00000024315 | + | - |  |
| ENSCAFG00000024414 | + | - | ENSG00000165349 |
| ENSCAFG00000024444 | + | - |  |
| ENSCAFG00000024527 | + | - | ENSG00000129749 |
| ENSCAFG00000025484 | + | - | ENSG00000165349 |
| ENSCAFG00000028875 | + | - | ENSG00000174807 |
| ENSCAFG00000028889 | + | - |  |
| ENSCAFG00000029008 | + | - | ENSG00000178586 |
| ENSCAFG00000029577 | + | - | ENSG00000159788 |
| ENSCAFG00000029978 | + | - | ENSG00000181927 |
| ENSCAFG00000030361 | + | - |  |
| ENSCAFG00000030488 | + | - | ENSG00000255251 |
| ENSCAFG00000030632 | + | - |  |
| ENSCAFG00000030713 | + | - | ENSG00000222009 |
| ENSCAFG00000030811 | + | - | ENSG00000182634 |
| ENSCAFG00000030983 | + | - |  |
| ENSCAFG00000031049 | + | - |  |
| ENSCAFG00000031636 | + | - | ENSG00000273703 |
| ENSCAFG00000031749 | + | - |  |
| ENSCAFG00000032054 | + | - | ENSG00000184956 |
| ENSCAFG00000032072 | + | - | ENSG00000258817 |
| ENSCAFG00000032183 | + | - | ENSG00000126821 |
| ENSCAFG00000032642 | + | - | ENSG00000221944 |
| ENSCAFG00000000178 | - | + | ENSG00000123384 |
| ENSCAFG00000000492 | - | + |  |
| ENSCAFG00000000749 | - | + |  |
| ENSCAFG00000001128 | - | + | ENSG00000042832 |
| ENSCAFG00000001378 | - | + | ENSG00000112062 |
| ENSCAFG00000001652 | - | + | ENSG00000188921 |
| ENSCAFG00000001857 | - | + | ENSG00000121766 |
| ENSCAFG00000002111 | - | + | ENSG00000146085 |
| ENSCAFG00000002509 | - | + | ENSG00000164010 |
| ENSCAFG00000003004 | - | + | ENSG00000131238 |
| ENSCAFG00000003568 | - | + | ENSG00000169169 |
| ENSCAFG00000003714 | - | + | ENSG00000137575 |
| ENSCAFG00000003818 | - | + |  |
| ENSCAFG00000004022 | - | + | ENSG00000138688 |
| ENSCAFG00000004655 | - | + | ENSG00000069399 |
| ENSCAFG00000004892 | - | + | ENSG00000160570 |
| ENSCAFG00000004931 | - | + | ENSG00000187091 |
| ENSCAFG00000005216 | - | + | ENSG00000168038 |
| ENSCAFG00000005286 | - | + | ENSG00000160460 |
| ENSCAFG00000005685 | - | + | ENSG00000101349 |
| ENSCAFG00000005717 | - | + | ENSG00000183091 |
| ENSCAFG00000006218 | - | + |  |
| ENSCAFG00000006295 | - | + | ENSG00000181023 |
| ENSCAFG00000006363 | - | + |  |
| ENSCAFG00000006607 | - | + | ENSG00000113657 |
| ENSCAFG00000006713 | - | + | ENSG00000105290 |
| ENSCAFG00000007251 | - | + | ENSG00000186081 |
| ENSCAFG00000007273 | - | + | ENSG00000083857 |
| ENSCAFG00000007597 | - | + | ENSG00000164163 |
| ENSCAFG00000009389 | - | + | ENSG00000010322 |
| ENSCAFG00000009410 | - | + | ENSG00000177054 |
| ENSCAFG00000009501 | - | + |  |
| ENSCAFG00000009811 | - | + | ENSG00000143061 |
| ENSCAFG00000009823 | - | + | ENSG00000080854 |
| ENSCAFG00000010828 | - | + | ENSG00000139438 |
| ENSCAFG00000010829 | - | + | ENSG00000163395 |
| ENSCAFG00000010840 | - | + | ENSG00000150594 |
| ENSCAFG00000011017 | - | + |  |
| ENSCAFG00000011630 | - | + | ENSG00000203710 |
| ENSCAFG00000011757 | - | + | ENSG00000180198 |
| ENSCAFG00000011819 | - | + | ENSG00000109927 |
| ENSCAFG00000012222 | - | + |  |
| ENSCAFG00000014025 | - | + | ENSG00000155657 |
| ENSCAFG00000014156 | - | + | ENSG00000161682 |
| ENSCAFG00000014622 | - | + | ENSG00000175920 |
| ENSCAFG00000014975 | - | + | ENSG00000075151 |
| ENSCAFG00000015124 | - | + | ENSG00000010292 |
| ENSCAFG00000015537 | - | + | ENSG00000102390 |
| ENSCAFG00000015747 | - | + | ENSG00000141503 |
| ENSCAFG00000016111 | - | + | ENSG00000164916 |
| ENSCAFG00000016358 | - | + | ENSG00000106009 |
| ENSCAFG00000017404 | - | + | ENSG00000121064 |
| ENSCAFG00000017714 | - | + | ENSG00000176438 |
| ENSCAFG00000018342 | - | + | ENSG00000185100 |
| ENSCAFG00000018846 | - | + | ENSG00000134278 |
| ENSCAFG00000019492 | - | + | ENSG00000130764 |
| ENSCAFG00000019783 | - | + | ENSG00000123454 |
| ENSCAFG00000020071 | - | + | ENSG00000065457 |
| ENSCAFG00000020189 | - | + | ENSG00000040199 |
| ENSCAFG00000023300 | - | + |  |
| ENSCAFG00000023904 | - | + | ENSG00000117971 |
| ENSCAFG00000024198 | - | + | ENSG00000248385 |
| ENSCAFG00000024996 | - | + |  |
| ENSCAFG00000025598 | - | + | ENSG00000136273 |
| ENSCAFG00000029079 | - | + | ENSG00000142556 |
| ENSCAFG00000029358 | - | + | ENSG00000131944 |
| ENSCAFG00000029380 | - | + | ENSG00000243509 |
| ENSCAFG00000029643 | - | + | ENSG00000142892 |
| ENSCAFG00000030771 | - | + | ENSG00000172954 |
| ENSCAFG00000030894 | - | + | ENSG00000270467 |
| ENSCAFG00000032185 | - | + | ENSG00000205495 |
| ENSCAFG00000032322 | - | + |  |
| ENSCAFG00000032578 | - | + | ENSG00000163395 |
